# Pharmacists’ views and reported practices in relation to a new generic drug substitution policy in Lebanon: a mixed methods study

**DOI:** 10.1186/s13012-017-0556-1

**Published:** 2017-02-17

**Authors:** Fadi El-Jardali, Racha Fadlallah, Rami Z. Morsi, Nour Hemadi, Mounir Al-Gibbawi, Magda Haj, Suzan Khalil, Youssef Saklawi, Diana Jamal, Elie A. Akl

**Affiliations:** 1 Department of Health Management and Policy, American University of Beirut, Beirut, Lebanon; 2 Center for Systematic Reviews of Health Policy and Systems Research (SPARK), American University of Beirut, Beirut, Lebanon; 3 Faculty of Medicine, American University of Beirut, Beirut, Lebanon; 4 Department of Internal Medicine, American University of Beirut, Beirut, Lebanon

## Abstract

**Background:**

Governments in both developed and developing countries have adopted generic drug substitution policies to decrease pharmaceutical expenditures and improve access to medicine. In August 2015, the Ministry of Public Health (MOPH) in Lebanon introduced generic drug substitution and a unified medical prescription form as policy instruments to promote generic drug use. The objective of this exploratory study was to examine the attitudes of community pharmacists and the reported practices in relation to the implementation of the new generic drug substitution policy.

**Methods:**

We used a cross-sectional mixed methods approach composed of self-administered questionnaires and semi-structured interviews. The study population consisted of community pharmacists in Lebanon. We randomly approached one pharmacy personnel from each selected community pharmacy. We conducted descriptive analyses to assess responses to questionnaire and regression analyses to understand associations between responses and respondent demographics. We analyzed qualitative data thematically.

**Results:**

Out of 204 invited community pharmacies, 153 pharmacies participated (75% response rate). The majority of respondents (64%) were in favor of generic drug substitution; however, less than half (40%) indicated they have substituted brand drugs for generic equivalents. Moreover, 57% indicated that the existing pricing system discourages them from performing generic drug substitution. Most respondents indicated that physicians are overusing the “non-substitutable” option (84%) and that there are technical problems with processing the new prescription form (78%). Less than half (47%) reported that the MOPH is performing regular audits on the forms collected by the pharmacy. While 45% of the respondents indicated that consumers have accepted most of the generic substitutions, 21% perceived the increase in generic drug dispensing to be significant. Findings suggested a potentially significant association between being informed about generic drugs and respondents’ support of the policy. Suggested strategies to address implementation challenges included strengthening stewardship function of MOPH, securing full commitment of health care providers, conducting educational and awareness campaigns about generic drugs and generic drug substitution, and aligning incentive systems of the key stakeholders.

**Conclusions:**

The majority of community pharmacists were supportive of generic drug substitution in general but not of the current implementation of the policy in Lebanon. Findings revealed implementation challenges at the provider, patient, and system level which are hindering attainment of the policy objectives. The key lessons derived from this study can be used for continuous improvement of the policy and its implementation.

**Electronic supplementary material:**

The online version of this article (doi:10.1186/s13012-017-0556-1) contains supplementary material, which is available to authorized users.

## Background

Ensuring access to medicines with assured quality, safety, and efficacy is considered a main component towards improved health outcomes [1]. According to estimates from the World Health Organization (WHO), the percentage of population without adequate access to essential medicines is less than 1% in high-income countries, 24% in middle-income countries, and 39% in low-income countries. This percentage rises to 50% in the poorest countries of Asia and Africa [2, 3].

One of the main barriers to access to medicine is their high costs [4, 5]. Generic medicines provide opportunities for major savings in healthcare expenditure due to their low price [6, 7]. Generic medicines are bioequivalent to their branded counterparts but are generally 20–90% less expensive [8–10]. The underuse of generics is considered as one of the leading causes of economic inefficiency in healthcare [11]. It has been estimated that switching procurement from branded drugs to the lowest-priced generic equivalents in the private sector in 17 developing countries could result in an average of 60% cost savings [12].

Governments in both developed and developing countries have introduced generic drug substitution (GS) policies to decrease pharmaceutical expenditures and improve access to medicine [6, 13]. Generic substitution allows pharmacists to dispense a generic drug containing the same active ingredient, form, dosage, and strength as the originator drug prescribed by the physician [14]. The laws governing generic substitutions vary from mandating pharmacists to substitute brand drugs with therapeutic equivalent generics unless prohibited by the prescriber (i.e., mandatory GS), to allowing pharmacists to perform generic substitution (i.e., permissive GS) and requiring patients’ consent before generic substitution [13]. Generic substitution is mandatory in six European countries (Sweden, Germany, Norway, France, Finland, and Spain) and permissive in others (Portugal, Denmark, Italy, Poland, Czech Republic, The Netherlands, Hungary, Poland, and Latvia) [15]. In the USA, generic substitution is a common practice with pharmacists substituting up to 84% of brand drug prescriptions for which generic equivalents are available [13].

### The Lebanese context

Pharmaceuticals account for over 25% of the total healthcare expenditure in Lebanon [16, 17] compared to an average of 17% in countries of the Organization for Economic Co-operation and Development (OECD) [18]. Lebanon’s per capita pharmaceutical spending is considered one of the highest in the Middle East [19]. In 2009, it was estimated that over 25% of household annual expenditures was spent on medications [17].

The pharmaceutical sector in Lebanon is dominated by imported medicines and patented brand names which constitute more than 80% of the total market share [17]. Around 40% of the Lebanese population have no form of health insurance coverage while the remaining 60% are insured either through public or private insurance. However, only around 20% of drugs consumed are thought to be reimbursed through one of the schemes [19].

Generic drug substitution has been proposed as a policy instrument in Lebanon to promote generic drug use and alleviate the extremely high cost of pharmaceuticals on households, government, and insurers. In March 2010, Parliament approved a law that provided pharmacists the right of substitution between a brand-name drug and a matching generic one under certain conditions (see Table 1). In 2011, the MOPH issued ministerial decision no. 1295, publishing the sample unified medical prescription form in three copies (one for the doctor, one for the pharmacist, and one for the patient) (see Table 1). However, the law was not officially implemented up until August 2015. The 4-year gap between its issuance and its implementation was a result of administrative hurdles and impediments from various stakeholders with commercial interests [20].

**Table 1:** Description of the generic drug substitution policy and the unified medical prescription form

	Characteristics
Generic drug substitution policy	The policy stipulated that pharmacists may disburse to bearers of prescription drugs generic medicines not mentioned in the prescription under the following conditions: •Alternative medicine should be included in the list of alternative medicines published by the MOPH in accordance with the WHO standards. •The medicine should contain the same active ingredient as the one mentioned in the prescription and in the same quantities and pharmacological form. •The price of sale of the substituted medicine in Lebanon should be less than the price of sale of the prescribed medicine. •The patient must agree to the substitution. •The physician must indicate acceptance of substitution. The pharmacist cannot change the prescription if the physician ticked the “Non-substitutable” (NS) box.
Unified medical prescription form	The old prescription form has been replaced with a new unified medical prescription form that permits generic drug substitution: •Patients, pharmacists, and health care providers are required to use one combined (i.e., unified) medical prescription form. •The form should be distributed in three copies: one for the physician, one for the patient, and one for the pharmacist. •The form is composed of several sections targeting physicians, pharmacists/radiologists/laboratory staff, insured patients, and insurance parties. •There is an option, in the form, that allows the physician to prescribe non-substitutable medications as per the 47th article of the practice law of pharmacy profession. •Physicians are required to purchase the form from the Order of Physician.

Assessing the implementation of a policy early on is critical as country experiences have shown that once policies are adopted, they are not always implemented as planned and do not necessarily achieve intended outcomes [21]. We are not aware of any study that has assessed the perception and reported practices of pharmacists with regards to implementation of the new generic drug substitution policy in Lebanon. A focus on pharmacists is critical given their strong influence on patients’ decisions and their pivotal role in the success of generic drug substitution policy [22–24]. Therefore, the objective of this exploratory study was to examine the attitudes of community pharmacists and the reported practices in relation to the implementation of the new generic drug substitution policy in Lebanon. It also aimed to gain a better understanding of emerging challenges as well as strategies that can be adopted to improve implementation of the policy.

## Method

### Study design and population

The study utilized a convergent mixed methods approach composed of both quantitative and qualitative components [25, 26]. Data collection was concurrent whereby respondents were first asked to complete a questionnaire followed by a semi-structured interview based upon their consent. The purpose of using a mixed methods approach was to expand on the ideas presented in the questionnaire to yield a more comprehensive understanding of the issue [27].

The study population consisted of licensed pharmacists and pharmacy technicians who work as frontline staff in community pharmacies in Lebanon. The sampling unit was community pharmacy, and the sampling frame was the list of 2806 licensed community pharmacies obtained from the Order of Pharmacists’ website.

For the quantitative component, the sample size was calculated based on 90% CI, a margin of error of 5%, and the assumption of 80% adherence rate to the rules that govern the unified medical prescription. This generated a sample size of 164 using an online sample size calculator [28]. A stratified convenience sampling method based on the six governorates (Beirut, Mount Lebanon, North Lebanon, Bekaa, South Lebanon, and Nabatiye) was used to select the pharmacies. To achieve this, we first stratified the pharmacies by governorate. We then selected a convenient sample of around 30 pharmacies from each governorate.

For the qualitative component, we invited respondents who completed the questionnaire to participate in semi-structured interviews. We opted to interview at least 5–6 pharmacists from each governorate.

### Survey instrument

The initial questionnaire was developed by the research team after an extensive review of the literature on generic drug substitution [24, 29–31]. Some of the items were adapted from existing measures whereas others, specifically those pertaining to the broader health system (e.g., receptiveness of MOPH), were created by the research team. The questionnaire was structured in line with the study objectives, taking into consideration the multitude of factors that may affect implementation of the new policy. We asked one community pharmacist, one professor of pharmacy, and one professor of pharmacology to review the questionnaire for face and content validity. We then pilot-tested the questionnaire with three community pharmacists (not involved in the actual study) and refined it based on their inputs. We originally developed the questionnaire in English and then translated it into Arabic language (which is the local language in Lebanon) by a professional translator. The questionnaire was back-translated by an independent researcher and any discrepancies were resolved through discussions with the core team. Both the English and Arabic versions were available, depending on participants’ preferences (see Additional file 1).

The final questionnaire consisted of the following five sections:

- Demographic and professional characteristics. These were measured by seven closed-ended questions and one open-ended question.
- Respondents’ attitudes towards generic drug substitution and the unified medical prescription. These were measured by three scales (each composed of several items): Attitude towards Generic Drug Substitution (two items), Attitude towards the Unified Medical Prescription (four items), and Layout/Structure of Form (two items). Each item was rated on a range from 1 to 5 where 1 indicates “Strongly disagree” and 5 indicates “Strongly agree.” Similar response scale and anchors were used elsewhere [32].
- Reported practices in relation to the implementation of the new generic drug substitution policy. These were measured by six scales (each composed of several items): Pharmacist Practices (five items); MOPH Responsiveness (four items); Functionality of Form (five items), Consumer Acceptance (four items); Outcome of Policy (five items); and Relation with Drug Companies (two items). Each item was rated on a range from 1 to 5 where 1 indicates “Strongly disagree” and 5 indicates “Strongly agree.”
- Perceived barriers to the implementation of the generic drug substitution policy. This was measured by one open-ended question.
- Suggested strategies to improve the implementation of the generic drug substitution policy. This was measured by one open-ended question.

The semi-structured interviews complemented and expanded on the questionnaire data by exploring participants’ experiences with implementing the new policy including perceived barriers and facilitators. The interviews were guided by an interview guide developed by the research team (see Additional file 1).

### Data collection

The study was approved by the Institutional Review Board at the American University of Beirut. Data was collected between January 2016 and March 2016. We randomly approached one pharmacy personnel from each selected community pharmacy. Whenever a selected pharmacy did not have a licensed pharmacist working as frontline staff, we sampled the pharmacy technician instead. Respondents were informed of the objective of the study and a verbal consent form was provided. Upon approval of respondents, a member of the research team provided the survey for self-completion; respondents were not asked to provide their names or that of their pharmacy to maintain confidentiality of responses. After survey completion, respondents were asked whether they were interested in taking part in the qualitative part.

The semi-structured interviews were conducted in the selected pharmacies by a member of the research team and lasted between 10 and 15 min each. The interviews were not audio-recorded; instead, thorough notes were taken by the interviewer. Interviews were conducted in Arabic and subsequently transcribed verbatim. All transcripts were translated to English by two members of the research team (RF and NH) and results were cross-validated.

### Data analysis

Data generated from the survey was entered and analyzed using statistical package SPSS 23.0 (*p* value = 0.05). To account for limited responses per category and facilitate interpretation, we grouped the responses for “Strongly agree” and “Agree” under the heading ”Strongly agree/Agree” and the responses for “Strongly disagree” and “Disagree” under the heading “Strongly disagree/Disagree”. The “Neutral” heading was retained. The percentage of positive responses for each item was calculated; negatively worded items were reversed when computing percent positive response (see Additional file 2 for results of the full Likert scale).

Scale scores were computed by summation of the item-based scores within the scale and dividing by the number of items with non-missing values. This generated a score that ranged from 1 to 5 for each scale, with higher scores signifying higher agreement to the item being assessed.

We used Cronbach’s alpha to measure the internal consistency and reliability of the scales. While Cronbach’s alpha values of 0.7 and above are recommended, a value of 0.6 or even lower can still be acceptable especially in exploratory studies and social sciences research [33, 34]. Bowling (1997) states that a value of 0.5 or above indicates good internal consistency [35], with lower values expected when using psychological constructs due to diversity of constructs being measured [36]. For the purpose of this exploratory study, items were considered to represent acceptable level of internal consistency if the Cronbach’s alpha value was 0.5 or above. Where applicable, we used the “alpha if item deleted” function to improve the internal consistency of scales while also ensuring there is no loss in criterion validity [37].

We used *T* tests to examine the association between the scales and the following variables: gender, profession, post-graduate qualification, ownership status of pharmacy, and whether or not the pharmacists received any teaching about generic versus brand-name drugs. We used ANOVA test to assess the association between the scales and the following variables: age, governorate, and country of education.

For the qualitative component, we conducted thematic analysis of the data generated from the interviews and the open-ended questions. We first coded the responses using an Excel spreadsheet. Then, two members of the research team (RF and NH) independently conducted open coding to segregate the findings into chunks that relate to similar concepts. This was followed by axial coding to organize emerging concepts into themes and subthemes that reflect study objectives and survey scales [38, 39]. They cross-checked and verified the identified themes.

## Results

### Quantitative results

#### Demographics and professional characteristics

Out of a total of 204 sampled pharmacies, 153 pharmacies had one individual agree to participate (75% response rate). The response rate by governorate is provided in Additional file 3. The majority of participants were licensed pharmacists (84.9%), between 20–40 years of age (69.6%), and have obtained their degrees from Lebanon (71.6%). Slightly, over half were males (51.4%) and owners of the pharmacy (56.2%). Around 75% reported receiving teachings about generic versus brand-name drugs. Only 38.7% of participants reported holding a postgraduate qualification (PharmD). Participants were roughly distributed across all six governorates, with Beirut (the capital city of Lebanon) having the highest number of participants. Table 2 shows the demographic and professional characteristics of respondents.

**Table 2:** Demographic and professional characteristics of respondents

Variable	Number	Percent^a^
Governorate		
Beirut	31	20
Bekaa	26	17
Mount Lebanon	23	15
North Lebanon	29	19
Nabatiye	21	14
South Lebanon	23	15
Gender		
Males	76	51
Females	72	49
Age range		
20–30 years	64	42
31–40 years	41	27
41–50 years	34	23
51–60 years	8	5
60+ years	4	3
Professional status		
Pharmacist	129	85
Pharmacy technician	23	15
Owner of pharmacy		
Yes	86	56
No	67	44
Country where degree was obtained		
Lebanon	106	72
Eastern Europe	20	14
Other (Libya, Russia, Syria, and Venezuela)	12	8
Western Europe	8	5
North America	2	1
Years of experience		
Less than 10 years	84	63
More than 10 years	49	37
Postgraduate qualification		
Yes	58	39
No	92	61
Teaching about generic versus brand-name drugs		
Yes	109	74
No	38	26

^a^All percentages have been rounded to one decimal point

#### Scale-based components

The Cronbach’s alpha value was above the cutoff value of 0.5 for all but three scales, indicating acceptable level of internal consistency. However, the value for each of the scales “Functionality of Form”, “Consumer Acceptance”, and “MOPH Responsiveness” was below 0.5, indicating poor level of internal consistency (see Table 3).

**Table 3:** Descriptive statistics and Cronbach’s Alpha on survey scales

Scales and corresponding items	Mean	Std. deviation	Strongly disagree/disagree *N* (%)^a^	Neutral *N* (%)^a^	Strongly agree/agree *N* (%)^a^
Attitude towards generic drug substitution (Cronbach’s alpha = 0.553)	3.78	0.85	49 (16)	35 (12)	219 (72)
1.I support generic substitution for all brand-name drugs for which generics are available.	3.55	1.01	30 (20)	25 (16)	97 (64)
2.It is acceptable that pharmacists perform generic substitution without obtaining permission from the prescribing physician.	4.01	1.03	19 (12)	10 (7)	122 (81)
Attitude towards unified medical prescription (Cronbach’s alpha = 0.529)	3.16	0.77	200 (33)	122 (20)	277 (46)
1.I support the implementation of the unified medical prescription.	2.94	1.22	58 (39)	36 (24)	55 (37)
2.The unified medical prescription helps promote the use of generic drugs in Lebanon.	3.09	1.25	53 (35)	31 (21)	66 (44)
3.The unified medical prescription helps identify physicians who are influenced by medical representatives.	3.89	1.09	20 (13)	22 (15)	107 (72)
4.The unified medical prescription helps regulate the pharmaceutical industry.	2.83	1.2	69 (46)	33 (22)	49 (32)
Layout/structure of form (Cronbach’s alpha = 0.705)	3.16	0.77	88 (29)	78 (26)	138 (45)
1.I am satisfied with the overall layout of the form.	2.89	1.09	57 (38)	40 (26)	55 (36)
2.The form is user-friendly.	3.32	1.01	31 (20)	38 (25)	83 (55)
Pharmacist practices (Cronbach’s alpha = 0.566)	3.30	0.64	186 (25)	193 (26)	376 (50)
1.Pharmacists are adhering to the laws that govern the unified medical prescription.	2.11	1.18	29 (19)	33 (22)	90 (59)
2.I have substituted brand drugs for generic equivalents in most of the prescriptions I have dispensed (excluding those prescriptions where the NS option was ticked).	3.04	1.14	53 (35)	38 (25)	60 (40)
3.I feel empowered to speak to patients about generic drug substitution since the implementation of the policy.	2.99	1.01	50 (33)	51 (34)	50 (33)
4.I sometimes consult with the physician when I feel the NS option is used unjustifiably.	3.32	1.18	42 (28)	27 (18)	82 (54)
5.I have been adhering to the Ministry of Public Health’s (MOPH) agreed list of substitutable generic drugs.	3.65	0.85	12 (8)	44 (29)	94 (63)
MOPH responsiveness (Cronbach’s alpha = 0.324)			117 (29)	173 (29)	311 (42)
1.The MOPH performs regular audits on the prescription forms collected by the pharmacy.	3.21	1.13	36 (24)	43 (29)	71 (47)
2.Generic drug equivalents approved by the MOPH are almost always in stock.	3.56	0.81	16 (11)	42 (28)	92 (61)
3.The MOPH’s national list of substitutable generic drugs is updated, accessible and easy to use.	3.18	1	38 (25)	51 (34)	63 (41)
4.The existing price structure discourages me from performing generic drug substitution.^b^	2.51	1.01	27 (18)	37 (25)	85 (57)
Functionality of form (Cronbach’s alpha = 0.452)			180 (75)	88 (12)	486 (13)
1.Physicians are adhering to the laws that govern the unified medical prescription form.	2.11	1.18	108 (72)	19 (13)	23 (15)
2.There are technical problems with the implementation of the unified medical prescription form.^b^	2.14	0.9	17 (11)	16 (11)	119 (78)
3.There are no clear guidelines on how to use the forms.^b^	2.36	1.06	27 (18)	21 (14)	104 (68)
4.Physicians in general are abusing the NS option.^b^	1.83	0.99	12 (8)	12 (8)	127 (84)
5.Some patients still show up with the old prescription form.^b^	2.03	1.08	16 (11)	20 (13)	113 (76)
Consumer acceptance (Cronbach’s alpha = 0.472)			168 (50)	130 (22)	305 (28)
1.Consumers generally express negative attitudes towards generic drugs.^b^	2.61	1.13	39 (26)	28 (19)	83 (55)
2.Consumers are not generally happy with the unified medical prescription.^b^	2.2	0.89	13 (9)	34 (22)	105 (69)
3.Consumers have been actively requesting generic substitutions for brand-name drugs.	2.89	1.06	62 (41)	39 (26)	50 (33)
4.Consumers have accepted most of the substitution suggestions offered by the pharmacy.	3.1	1.06	54 (36)	29 (19)	67 (45)
Relation with drug companies (Cronbach’s alpha = 0.629)	2.76	0.92	140 (47)	68 (23)	92 (31)
1.My drug substitution choice is influenced by information from medical drug representatives.	2.6	1.01	79 (52)	36 (24)	36 (24)
2.It is acceptable that pharmacists rely on medical drug representatives to learn about alternative drug substitutions.	2.91	1.15	61 (41)	32 (21)	56 (38)
Outcome of the policy (Cronbach’s alpha = 0.511)	2.67	0.61	299 (50)	188 (25)	271 (25)
1.The number of generic dispensing at my pharmacy has increased considerably since the implementation of the policy.	2.59	0.97	81 (53)	39 (26)	32 (21)
2.There was an initial peak in generic dispensing following the implementation of the policy that was subsequently attenuated.	2.72	1.02	73 (48)	39 (26)	40 (26)
3.The overall patient expenditure on medicine has decreased since the implementation of the policy.	2.86	1.09	69 (45)	31 (20)	52 (34)
4.There has been a noticeable shift in pharmaceutical companies’ promotional strategies targeting pharmacists.	2.97	1.05	53 (35)	53 (35)	46 (30)
5.The policy is creating conflicts between the physician, the pharmacist, and the patient.^b^	3.77	1.09	23 (15)	26 (17)	101 (67)

^a^All percentages have been rounded to the nearest one

^b^Reverse scores

The mean scores computed for the different scales varied between 2.47 and 3.78 (on a range from 1 to 5). “Attitudes towards Generic Drug Substitution” had the highest mean score (3.78), indicating higher average agreement to the items being assessed whereas “Functionality of Form” (2.47) had the lowest score, reflecting poor average agreement to the items being assessed (see Table 3). The details of each scale and corresponding items are further discussed below. We grouped the scales into those assessing respondents’ attitudes and those assessing reported practices, respectively.

#### Respondents’ attitudes towards generic substitution and the unified medical prescription

Respondents’ attitudes were assessed using the following three scales: “Attitude towards Generic Drug Substitution”, “Attitude towards the Unified Medical Prescription”, and “Layout of Form”.

Respondents expressed positive attitude towards generic drug substitution in general, with 64% supporting generic drug substitution for all brand-name drugs for which generics are available. In addition, it was acceptable for 81% of respondents that pharmacists perform generic substitution without obtaining permission from the prescribing physician. On the contrary, respondents expressed less positive attitudes towards the unified medical prescription, with only 37% supporting its current implementation in Lebanon. Furthermore, less than half of respondents agreed with the statements that it promotes the use of generic drugs in Lebanon (44%) or that it helps regulate the pharmaceutical industry (32%). Respondents who indicated receiving teachings about generic versus brand-name drugs had significantly higher mean scores regarding attitudes towards the unified medical prescription (*p* = 0.035) (see Additional file 4). With regards to the layout of the form, 55% of the respondents agreed that the form is user-friendly and 36% indicated that that they are satisfied with its overall layout.

#### Reported practices in relation to the New Generic Drug Substitution Policy

Reported practices were assessed using six scales: “Pharmacist Practice”, “MOPH responsiveness”, “Functionality of Form”, “Consumer Acceptance”, “Relations with Pharmaceutical Industry”, and “Outcome of Policy”.

When it comes to pharmacists’ practices, over half of the respondents indicated that pharmacists are adhering to the laws that govern the unified medical prescription (59%). Similarly, 63% agreed that they are adhering to the MOPH’s list of substitutable generic drugs. Nonetheless, only 40% reported that they have substituted brand drugs for generic equivalents in most of the prescriptions they have dispensed for which substitution was allowed. Furthermore, 33% agreed that they felt more empowered to speak to patients about generic drug substitution since implementation of the policy (see Table 3). Respondents who indicated receiving teachings about generic versus brand-name drugs had significantly higher mean scores regarding pharmacist practices (*p* < 0.001). On the contrary, significantly lower mean scores were observed among respondents reporting less than 10 years of experiences (*p* = 0.049) (see Additional file 4). With regards to their relations with pharmaceutical companies, 24% of respondents agreed with the statement that their drug substitution choice is influenced by information from medical drug representatives.

When asked about MOPH responsiveness to the new policy, 47% of the respondents indicated that the MOPH is performing regular audits on the unified medical prescription forms. In addition, 41% agreed that the national list of substitutable generic drugs is updated, accessible, and easy to use. Furthermore, 57% indicated that the existing pricing system set by the MOPH discourages them from performing generic drug substitution. Those who reported having less than 10 years of experience had significantly lower mean scores regarding MOPH responsiveness to the policy (*p* = 0.009).

With regards to functionality of the unified medical prescription form, the majority of respondents agreed with the statements that physicians are overusing the “non-substitutable” option (84%) and that they are not adhering to the laws that govern the unified medical prescription (72%). Most respondents also indicated that there are technical problems related to processing the forms (78%), with no clear guidelines on how to use them (68%). Furthermore, 76% indicated that consumers still show up to the pharmacy with the old prescription form. As for consumer acceptance, the majority of respondents agreed with the statements that consumers have expressed negative attitudes towards generic drugs (55%) and the unified medical prescription (69%). In addition, 45% reported that consumers have accepted most of the generic drug substitutions, and one-third indicated that consumers have actively requested generic substitution for brand-name drugs.

The questionnaire also attempted to assess the outcome of the new policy. Less than a quarter of respondents (21%) indicated that the number of generic dispensing at their pharmacies has increased considerably after implementation of the policy. In addition, around one-third (34%) indicated that the overall patient expenditure on medicine has decreased after its implementation. Furthermore, 30% agreed that there has been a noticeable shift in pharmaceutical companies’ promotional strategies targeting pharmacists after implementation of the policy.

Ownership of pharmacy, age, professional status, and country of degree were not found to be significantly associated with any of the scales (see Additional file 4).

### Qualitative results

Seventy-five and 55 individuals, respectively, completed the first and second open-ended question. In addition, 29 individuals from all but one governorate (Nabatiye) participated in the semi-structured interviews. The emerging themes were grouped under the following two main domains: barriers to implementation of the generic drug substitution policy and strategies to facilitate its implementation, respectively. Additional file 5 summarizes the emerging themes in tabular format, categorized into patient, provider, organizational, and systems level.

#### Perceived barriers to implementation of the policy

An overarching barrier was the lack of preparedness of end users to implement generic drug substitution policy, with subsequent poor adherence and lack of clarity on the purpose, rights, and expectations from each party.

The overuse of the “non-substitutable (NS)” option for unjustified reasons was highlighted as a main obstacle hindering generic drug substitution. This has been exacerbated by the absence of mechanisms to monitor the prescribing patterns of physicians. As stated by one participant:

*“The policy did nothing; doctors found their way around it by marking ‘NS’ in most prescriptions, even for those where another equivalent medication exists with a lower price”*—[Interviewee 15; female pharmacist; Beirut]

Another frequently reported challenge is the lack of adherence of some physicians to the policy. This is manifested in their continued use of the old prescription form specifically with patients who are uninsured or who have private insurance. In addition, some physicians have been charging patients for the new form as a way of shifting the cost burden to them, as highlighted by one participant:

*“The price of the form is problematic; some physicians are charging patients up to 20,000 [Lebanese Pounds] for the form”* (US$1 = 1506.5 Lebanese Pound (LBP))—[Interviewee 8; male pharmacist; Mount Lebanon]

Furthermore, participants reported that dispensaries and governmental hospitals were still using the old prescription form. This, in turn, has serious implications as patients coming from these settings are the most in need of non-expensive drugs and thus could benefit from the new prescription form which permits generic substitution.

Physicians’ relationship with the pharmaceutical industry was another perceived factor hampering proper implementation of the generic drug substitution policy. According to participants, this has prompted some physicians to persuade patients into refusing generic substitutions offered by pharmacists.

Another recurrent barrier related to insufficient policy support from pharmacists themselves due to lower profitability, increased administrative burden, resistance from consumers, and a perceived sense of “disrespect to the pharmacy profession as a whole, due to the “NS” option”. In addition, a number of pharmacists stated that they still accept the old prescription form for the purpose of customer retention, as illustrated below:

*“I have been losing customers because some doctors are writing on a white paper [instead of the new prescription form] and I’m not accepting it while other pharmacies are…”*—[Interviewee 3; male pharmacist; North Lebanon]

Participants frequently mentioned that the existing pricing system also impacts the success of the generic drug substitution policy according to the following scenario: the pricing strategy adopted by the MOPH prior to the introduction of the new policy had led to a significant reduction in the prices of brand-name drugs. With the launching of the unified medical prescription, several pharmaceutical companies were prompted to further lower the prices of their patented drugs to compete with generics. Consequently, the prices of some brand drugs became similar to, and in some cases, lower than those of their generic equivalents. According to participants, this scenario undermined the purpose of the policy to promote generic drugs in the first place. This has been further exacerbated by the absence of an incentive system to encourage pharmacists to perform generic substitutions.

Additional reported barriers included insufficient awareness and education of providers, patients and third-party insurers about generic drugs, and the new drug substitution policy as well as poor trust in the quality of generics in the market. Some participants also pointed to administrative hurdles related to processing of bills by third party payers for drugs disbursed according to the new prescription form (see Additional file 5).

#### Suggested strategies to improve implementation of the policy

Securing the full commitment of physicians was repeatedly suggested as crucial for successful implementation of the generic drug substitution policy. Whereas a number of participants supported the complete elimination of the “NS” option from the form, the majority preferred the establishment of mechanisms to discourage its overuse such as a minimum permissible number of NS that can be marked by a physician per month for different classes of drugs. One participant also suggested computerizing the medical prescription form to strengthen the implementation process.

A number of participants indicated that the MOPH should conduct periodic monitoring and inspections, primarily of physicians and dispensaries, as well as enforce legal and non-legal measures on entities that violate implementation of the policy. They also pointed to the need to ensure the quality of generic drugs circulating the market, including strengthening the registration procedure and mandating quality and bioequivalence testing of drugs entering the market. Some participants also highlighted the need for educational and awareness campaigns about generic drugs in general and the unified medical prescription in particular. They also stressed on the importance of establishing incentive systems to encourage generic drug substitution.

Participants also pointed to the need to reduce administrative burdens associated with processing the form due to inconveniences and delays in treatment associated with sending a patient or even a family member back to the physician to correct or complete the prescription form. Additional highlighted strategies included: subsidizing or providing free consultation visits to poor patients so that they can afford such visits whenever they need a medication (particularly for refill prescriptions), establishing appropriate pricing policies that create a competitive market for generic drugs, and regulating interactions between healthcare providers and drug companies (see Additional file 5).

## Discussion

### Summary and interpretation of findings

The findings from this study revealed that the majority of pharmacists supported generic drug substitution in general. In spite of this favorable attitude, respondents were not fully supportive of the current implementation of the policy in Lebanon. While the main aim of this policy instrument was to reduce medicine expenditures by promoting generic drugs, findings revealed that pharmacists did not perceive the increase in generic drug dispensing or the decrease in overall patient expenditures to be significant. That being said, pharmacists believed that the policy had an important influence on the pricing strategies of some pharmaceutical companies by pressuring them to lower the prices of their brand-name drugs to compete with generic equivalents. On the one hand, this makes competition policy a potentially important policy option in LMICs [6]; on the other hand, and as reported by respondents, it may also discourage generic substitution for those drugs whose prices have become similar to their generic equivalents. This, in turn, highlights the importance of establishing appropriate medicine prices and pricing systems to promote generic drugs [40].

The findings also pointed to poor adherence of the key stakeholders including physicians, pharmacists, and patients to the policy. The majority of respondents indicated that physicians and dispensaries have not been adhering to the new form with continued use of the old form, especially among patients who are uninsured or who have private insurance. This has financial and health implications given that almost half the population in Lebanon lacks insurance coverage [17]. Where the new prescription form is being utilized, a significant number of physicians seem to be overusing the NS option. One reason for this could relate to unethical promotional incentives by pharmaceutical industries. Furthermore, some physicians are reportedly charging patients for the form, which represents an additional burden for them. As for pharmacists themselves, they have admitted to accepting the old form due to fear of losing customers to other pharmacies. Furthermore, they have refrained from performing generic substitution for many prescriptions that allowed substitution; a frequently reported reason related to the poor incentive system in place to encourage generic substitution. With regards to consumers, they have reportedly demonstrated poor acceptance of generic drugs and generic drug substitution, possibly due to poor knowledge of generic drug substitution, and a preference for drugs prescribed by physicians. Findings suggested a potentially significant association between being informed about generic drugs and respondents’ support of the generic substitution policy.

Perspectives in the literature on the key components of successful policy implementation have been summarized in a framework encompassing seven dimensions: the policy, its formulation, and dissemination; social, political, and economic context; leadership for policy implementation; stakeholder involvement in policy implementation; implementation planning and resource mobilization; operations and services; and feedback on progress and results [23]. By matching the challenges reported in our study to this framework, it becomes evident that the policy implementation in Lebanon has been challenged by insufficient dissemination and understanding of the policy by those responsible for and affected by its implementation; insufficient attention to the socio-economic factors outside the policy process; lack of strong leadership to ensure follow-through, resources, and accountability for translating the policy into practice; poor coordination mechanisms and commitments of individuals and organizations responsible for delivering the services outlined in the policy; and weak capacity of the MOPH to regularly monitor, collect, disseminate, and use feedback to evaluate progress towards attainment of the policy goals.

### Comparison to findings from other studies

Similar to this study, findings from studies conducted in Malaysia, New Zealand, Czechia, and Saudi Arabia found that pharmacists support generic drug substitution in general [23, 30, 32, 41]. However, contrary to our finding, the majority of pharmacists in Iran (71.6%) and the USA (83.8%) stated that they have generically substituted prescriptions that allowed generic substitution [42, 43]. While this may reflect obstacles in implementation of the policy in Lebanon, it is also important to take into consideration that the policy is still in its first year of implementation.

Contrary to our findings, a study conducted in Australia found that less than 20% of prescriptions dispensed had substitution prohibited by the physician [44]. Although the NS option may be used for drugs with narrow therapeutic indices [43, 45], it is estimated that the maximum prescription rate that can be filled by generic drugs can reach up to 80% [46].

Challenges reported in this study paralleled those reported in other studies. These included poor adherence of physicians and pharmacists to the new policy [45], lack of trust of health professionals and consumers in quality and bioequivalence of drugs [47–49], incentives from pharmaceutical companies [50], and absence of appropriate medicine prices and pricing systems to promote generic medicine [45, 48]. Findings on the key enablers for promoting generic drug substitution confirmed those reported previously and included the presence of supportive legislation and regulations to promote a competitive market for generic drugs, creation of a trusted medicines regulatory authority, quality assurance capacity, acceptance by health professional and the public, and pro-generic drug incentives for prescribers, dispensers and patients [6, 13, 32, 43].

### Strengths and limitations

To our knowledge, this is the first study to assess the implementation of the generic drug substitution policy in Lebanon. The study is timely as it constitutes a baseline assessment of the policy following its early implementation. In addition, data triangulation through the use of surveys and semi-structured interviews strengthened the internal validity of our findings. Furthermore, the survey achieved an overall response rate of 75%, which is considered acceptable.

The study has a number of limitations that should be taken into consideration when interpreting the results. First, the findings on current practices are based on the perception of pharmacists, and not on objective data, such as prescription records at pharmacies. However, as prescription records are not readily available or accessible in Lebanon, we employed data triangulation to help validate the findings. Another limitation relates to the questionnaire where the Cronbach alpha’s score was below 0.5 for three scales. Since discarding some of the items using the “alpha if item deleted” function did not significantly alter the Cronbach’s alpha coefficients, it is possible to attribute the low internal consistencies for these constructs to the small number of items (fewer than 10) included in the scales [51]. Future surveys should consider revising some of the survey questions to enhance scale reliability. A further limitation is the cross-sectional study design which reflects only one point in time and therefore may not capture changes in respondents’ attitudes and practices over time. While these studies cannot ascertain a causality relationship for any observed association, they can still provide useful information on magnitudes, patterns, and trends which can help inform proper implementation. Finally, it is important to note that we could not evaluate the representativeness of the respondent demographics due to the absence of a national data on characteristics of community pharmacists. However, we believe that this sample is likely to be representative. First, we sampled pharmacists from all six provinces in Lebanon, which enhanced the geographical representativeness of the findings. Second, a study of the pharmacy manpower in Lebanon found that newly graduated (and young) pharmacists were more likely to work in community pharmacists [52]; thus, we can deduce that a response bias was unlikely to have occurred.

### Implication for policy and practice

As a lead entity in Lebanon, the MOPH should play a key role in coordination and oversight of the generic drug substitution policy, monitor the extent of implementation and achievement of targets, and establish consequences for non-compliance. These should be complemented by supporting policies to ensure appropriate pricing and purchasing strategies as well as regulate interactions of health care provider with pharmaceutical industries. Additional efforts should be invested in publicizing the national substitution drug list as well as issuing a full list of over-the-counter drugs that can be disbursed by pharmacists without a prescription form. Professional associations should also play a leading role in ensuring their respective health care professionals are educated about the purpose of the policy and are committed to its proper implementation.

The establishment and alignment of stakeholder incentives is another critical area worth considering. The MOPH could consider establishing quality and accreditation standards for pharmacies and ensure generic substitution goals and indicators are integrated as part of the remuneration system for pharmacists [53, 54]. As for physicians, prescribing targets for generic drugs could be established and linked to financial incentives and performance appraisals [48, 54]. Considerations could also be given to establish limits on the number of times the NS option can be marked by a physician per month for certain drug classes. Professional acceptance of the policy can further be facilitated by utilizing generic names in clinical guidelines and formularies and providing a cross-reference list of brand and generic names for all prescribers [55]. As for patients, subsidizing consultation visits, particularly for the poor and those with refill prescriptions, could be considered.

Another important area relates to strengthening pharmaceutical regulations to ensure quality and availability of generic medicines [56]. This could be complemented by media campaigns to raise awareness and educate health care professionals and the general public about generic drugs and demystify misconceptions about inferiority of generics relative to branded drugs [14]. Equally important is the need to incorporate courses on generic drugs in the undergraduate curricula of pharmacists to help support generic substitution and implementation of the new policy [57, 58].

Finally, to ensure sustainability, it is critical to establish measurable indicators for ongoing monitoring and evaluation at all stages of policy implementation [40]. Computerization of the unified medical prescription form at later stages can facilitate ongoing and timely surveillance as well as alleviate reported administrative hurdles related to processing the form.

### Implications for research

It is important to follow up on the policy implementation in the upcoming years, using more robust studies. These would assess more objective outcomes (e.g., actual as opposed to reported practices) and use better methodological designs such as interrupted time series or pre–post studies. Additional research is needed on the practices of hospital pharmacists given the different regulatory measures employed in such context. Future research could also explore physicians’ and consumers’ perceptions and practices in relation to the implementation of the generic drug substitution policy.

## Conclusions

The majority of community pharmacists were supportive of generic drug substitution in general but not of the current implementation of the policy in Lebanon. Although the policy may be well intentioned at its core, the realization of its objectives has been undermined by weaknesses in implementation of the unified medical prescription form and absence of supporting policies and incentive systems to minimize perverse behaviors. Securing the full commitment of pharmacists and physicians, strengthening the stewardship function of the MOPH, and establishing pro-generic drug incentives are crucial for successful implementation of the policy. The key lessons derived from this study can be used for continuous improvement of the policy and its implementation.

## Additional files

Additional file 1: Survey instrument. (PDF 898 kb)
Additional file 2: Results of the full Likert scale. (PDF 275 kb)
Additional file 3: Questionnaire distribution by governorate and response rates. (PDF 84 kb)
Additional file 4: ANOVA and *T* tests results. (PDF 314 kb)
Additional file 5: Barriers and strategies to facilitate implementation of the generic drug substitution policy, as perceived by respondents. (PDF 193 kb)

## Abbreviations

GS: Generic substitution
LMICs: low- and middle- income countries
MOPH: Ministry of Public Health
NS: Non-substitutable
WHO: World Health Organization

## References

[1] World Health Organization (WHO). Everybody’s business--strengthening health systems to improve health outcomes: WHO’s framework for action. 2007. http://www.who.int/healthsystems/strategy/everybodys_business.pdf?ua=1. Accessed 15 Apr 2015.

[2] Hogerzeil HV, Mirza Z. The world medicines situation 2011: access to essential medicines as part of the right to health. Geneva: World Health Organization; 2011.

[3] World Health Organization (WHO) (2004). The world medicines situation.

[4] Cameron A (2009). Medicine prices, availability, and affordability in 36 developing and middle-income countries: a secondary analysis. Lancet.

[5] Huskamp HA (2003). The effect of incentive-based formularies on prescription-drug utilization and spending. N Engl J Med.

[6] Kaplan WA (2012). Policies to promote use of generic medicines in low and middle income countries: a review of published literature, 2000–2010. Health Policy.

[7] Kaplan WA, Wirtz VJ, Stephens P (2013). The market dynamics of generic medicines in the private sector of 19 low and middle income countries between 2001 and 2011: a descriptive time series analysis. PLoS One.

[8] King DR, Kanavos P (2002). Encouraging the use of generic medicines: implications for transition economies. Croat Med J.

[9] Generic Pharmaceutical Association. Generic drug savings in the US (fourth annual edition: 2012). 2012. http://www.gphaonline.org/media/cms/IMSStudyAug2012WEB.pdf. Accessed 15 Sept 2015.

[10] Food and Drug Administration (FDA) (2015). What are generic drugs?.

[11] World Health Organisation (WHO). Access to new medicines in Europe: technical review of policy initiatives and opportunities for collaboration and research [Internet]. 2015. http://www.euro.who.int/__data/assets/pdf_file/0008/306179/Access-new-medicines-TR-PIO-collaboration-research.pdf?ua=1. Accessed 15 May 2015.

[12] Cameron A (2012). Switching from originator brand medicines to generic equivalents in selected developing countries: how much could be saved?. Value Health.

[13] Hassali MA (2014). The experiences of implementing generic medicine policy in eight countries: a review and recommendations for a successful promotion of generic medicine use. Saudi Pharm J.

[14] Dylst P (2013). Generic medicines: solutions for a sustainable drug market?. Appl Health Econ Health Policy.

[15] European Generic Medicines Association (2011). Market review.

[16] Ammar W (2009). Health beyond politics.

[17] Blominvest Bank (2016). Pharmaceuticals and healthcare in Lebanon: numerous opportunities to explore.

[18] Organization for Economic Cooperation and Development (OECD). Health at a glance 2013: OECD indicators. OECD publishing; 2013.

[19] The Lebanon brief. 2013. http://www.databank.com.lb/docs/Education%20-%20blominvest%20oct%202013.pdf]. Accessed 13 Mar 2015.

[20] Arbid J. Waiting for (re)forms. Executive Magazine. 2015. http://www.executive-magazine.com/economics-policy/healthcare-waiting-for-reforms. Accessed 17 Oct 2015.

[21] Bhuyan A, Jorgensen A, S. Sharma S (2010). Taking the pulse of policy: the policy implementation assessment tool.

[22] Palagyi M, Lassanova M (2008). Patients attitudes towards experience with use of generics in Slovakia, performance of generic substitution. Bratisl Lek Listy.

[23] Chong CP (2011). A nationwide study on generic medicines substitution practices of Australian community pharmacists and patient acceptance. Health Policy.

[24] Chong CP (2010). Evaluating community pharmacists’ perceptions of future generic substitution policy implementation: a national survey from Malaysia. Health Policy.

[25] Tashakkori A, & Teddlie C. Mixed methodology: combining qualitative and quantitative approaches (Vol. 46). Thousand Oaks: Sage; 1998.

[26] Creswell JW, Plano Clark VL, Gutmann ML, Hanson WE, Tashakkori A, Teddlie C (2003). Advances in mixed methods research designs. Handbook of mixed methods in social and behavioral research.

[27] Morse JM (1991). Approaches to qualitative-quantitative methodological triangulation. Nurs Res.

[28] Raosoft, Inc (2004). Sample size calculator.

[29] Babar ZU (2011). Evaluating pharmacists’ views, knowledge, and perception regarding generic medicines in New Zealand. Res Social Adm Pharm.

[30] Maly J (2013). Analysis of pharmacists’ opinions, attitudes and experiences with generic drugs and generic substitution in the Czech Republic. Acta Pol Pharm.

[31] Toklu HZ (2012). Knowledge and attitudes of the pharmacists, prescribers and patients towards generic drug use in Istanbul–Turkey. Pharm Pract (Granada).

[32] Alkhuzaee FS, Almalki HM, Attar AY, Althubiani SI, Almuallim WA, Cheema E, Hadi MA (2016). Evaluating community pharmacists’ perspectives and practices concerning generic medicines substitution in Saudi Arabia: a cross-sectional study. Health Policy.

[33] Hair JF, Anderson RE, Babin BJ, Black WC. Multivariate data analysis: A global perspective. Upper Saddle River: Pearson; 2010.

[34] Aron A, Aron E (1999). Statistics for psychology.

[35] Bowling A (1997). Research methods in health.

[36] Field A (2009). Discovering statistics using SPSS (and sex and drugs and rock ‘n’ roll).

[37] Gliem JA, Gliem RR. Calculating, interpreting, and reporting Cronbach’s alpha reliability coefficient for Likert-type scales. Midwest Research-to-Practice Conference in Adult, Continuing, and Community Education. 2003.

[38] Kendall J (1999). Axial coding and the grounded theory controversy. West J Nurs Res.

[39] LaRossa R (2005). Grounded theory methods and qualitative family research. J Marriage Fam.

[40] Nguyen TA (2015). Policy options for pharmaceutical pricing and purchasing: issues for low- and middle-income countries. Health Policy Plan.

[41] Babar ZU (2007). Evaluating drug prices, availability, affordability, and price components: implications for access to drugs in Malaysia. PLoS Med.

[42] Yousefi N, Mehralian G, Peiravian F, Noee F (2015). Generic substitution policy implementation: a pharmacists’ perspective survey.

[43] Mott DA, Cline RR (2002). Exploring generic drug use behavior: the role of prescribers and pharmacists in the opportunity for generic drug use and generic substitution. Med Care.

[44] Kalisch LM, Roughead EE, Gilbert AL (2007). Pharmaceutical brand substitution in Australia—are there multiple switches per prescription?. Aust N Z J Public Health.

[45] O’Leary A (2015). Generic medicines and generic substitution: contrasting perspectives of stakeholders in Ireland. BMC Res Notes.

[46] Ess SM, Schneeweiss S, Szucs TD (2003). European healthcare policies for controlling drug expenditure. Pharmacoecon.

[47] Colgan S (2015). Perceptions of generic medication in the general population, doctors and pharmacists: a systematic review. BMJ Open.

[48] Godman B (2009). Multifaceted national and regional drug reforms and initiatives in ambulatory care in Sweden: global relevance. Expert Rev Pharmacoecon Outcomes Res.

[49] Awaisu A (2014). Knowledge, attitudes, and practices of community pharmacists on generic medicines in Qatar. Int J Clin Pharm.

[50] El-Dahiyat F, Kayyali R (2013). Evaluating patients’ perceptions regarding generic medicines in Jordan. J Pharm Policy Pract.

[51] Lowenthal KM (2001). An introduction to psychological tests and scales.

[52] Salameh P, Hamdan I (2007). Pharmacy manpower in Lebanon: an exploratory look at work-related satisfaction. Res Soc Adm Pharm.

[53] Dylst P, Vulto A, Simoens S (2012). How can pharmacist remuneration systems in Europe contribute to generic medicine dispensing?. Pharm Pract (Granada).

[54] Sermet C (2010). Ongoing pharmaceutical reforms in France: implications for key stakeholder groups. Appl Health Econ Health Policy.

[55] Schaefer K (2007). Changing GPs’ prescription patterns through guidelines and feedback. InterventStudy Pharmacoepidemiol Drug Saf.

[56] World Health Organization (2001). How to develop and implement a national drug policy.

[57] Al-Tamimi SK (2016). The need to incorporate generic medicines topic in the curriculum of Yemeni pharmacy colleges. Int J Pharm Pract.

[58] Alrasheedy AA, Hassali MA, Aljadhey H, Al-Tamimi SK (2014). The need to cover generic medications and generic substitution practice in the curricula of pharmacy colleges in Saudi Arabia. Am J Pharm Educ.

