# Supplementary material for: Pharmacists’ views and reported practices in relation to a new generic drug substitution policy in Lebanon: a mixed methods study
Source: Implement Sci. 2017 Feb 17;12:23. doi: 10.1186/s13012-017-0556-1 (PMC5316154; doi:10.1186/s13012-017-0556-1)
Supplement: Additional file 1: — Survey instrument. (PDF 898 kb) [file 13012_2017_556_MOESM1_ESM.pdf]

Additional file 1: Survey Instrument

Serial number: _____

Questionnaire

Thank you for taking part in this survey. The survey is divided into four short sections and is expected to take no longer than 20 minutes to complete.
You are not required to provide your name or signature in any part of the survey.

Section I- Demographic characteristics

Please fill in the required information by placing an "x" on the most accurate selection (choose only one)

Variables	
**Kaza**	☐ Beirut,<br>☐ Mount Lebanon,<br>☐ North Lebanon,<br>☐ Beqaa,<br>☐ South Lebanon,<br>☐ Nabatiye
**Gender**	☐ Male<br>☐ Female
**Age Range**	☐ 20-30<br>☐ 31-40<br>☐ 41-50<br>☐ 51-60<br>☐ >60
**Professional status**	☐ Pharmacist<br>☐ Pharmacy technician<br>☐ Other (please specify):<br>________________________________
**Owner of the pharmacy**	☐ Yes<br>☐ No
**Country/region where Degree was obtained**	☐ Lebanon<br>☐ North America<br>☐ Western Europe<br>☐ Eastern Europe (including Russia)<br>☐ Other (please specify country):<br>_______________________________
**Postgraduate Qualification (PharmD)**	☐ Yes<br>☐ No
**Number of years of work experience as a pharmacist or pharmacy technician**	Please indicate here: _____________
**Have you received any teaching in your studies about generic versus brand name drugs?**	☐ Yes<br>☐ No

<u>Section II: Attitude towards generic drug substitution and the unified medical prescription</u>

In this survey, "brand" refers to a brand drug while 'generic' refers to the therapeutic equivalent generic

Please indicate your degree of agreement with the following statements by placing an "X" on the most accurate selection:

**Statement**	**Strongly disagree**	**Disagree**	**Neutral**	**Agree**	**Strongly agree**
1. I support generic substitution for all brand name drugs for which generics are available.					
2. It is acceptable that pharmacists perform generic substitution without obtaining permission from the prescribing physician.					
3. It is acceptable that pharmacists rely on medical drug representatives to learn about alternative drug substitutions.					
4. I support the implementation of the unified medical prescription.					
5. The unified medical prescription helps promote the use of generic drugs in Lebanon.					
6. The unified medical prescription helps identify physicians who are influenced by medical representatives.					
7. The unified medical prescription helps regulate the pharmaceutical industry.					
8. The "non- substitutable (NS)" option, which prohibits pharmacists from changing the prescription if ticked by physicians, should be kept in the form.					
9. I am satisfied with the overall layout of the form.					
10. The form is user-friendly.					

<u>Section III: Reported practices in relation to the implementation of the new generic drug substitution policy</u>

Please indicate your degree of agreement with the following statements by placing an “X” on the most accurate selection:

Statement	Strongly disagree	Disagree	Neutral	Agree	Strongly agree
1. Pharmacists are adhering to the laws that govern the unified medical prescription form.					
2. I have substituted brand drugs for generic equivalents in most of the prescriptions I have dispensed (excluding those prescriptions where the “NS” option was ticked).					
3. The existing price structure discourages me from performing generic drug substitution.					
4. My decision to substitute a drug is influenced by the socio-economic status of the patient.					
5. My drug substitution choice is influenced by information from medical drug representatives.					
6. I feel empowered to speak to patients about generic drug substitution since the implementation of the policy.					
7. I don’t offer generic substitution unless it is specifically requested by the patient.					
8. I sometimes consult with the physician when I feel the “NS” option is used unjustifiably.					
9. I have been adhering to the MOPH’s agreed list of substitutable generic drugs.					
10. Physicians are adhering to the laws that govern the unified medical prescription form.					
11. There are technical problems with the implementation of the unified medical prescription form.					
12. There are no clear guidelines on how to use the different forms.					
13. Physicians in general are abusing the “NS” option.					
14. The Ministry of Public Health (MOPH) performs regular audits on the prescription forms collected by the pharmacy.					
15. Generic drug equivalents approved by the MOPH are almost always in stock.					
16. The MOPH’s national list of substitutable generic drugs is updated, accessible and easy to use.					

Statement	Strongly disagree	Disagree	Neutral	Agree	Strongly agree
17. Consumers generally express negative attitudes towards generic drugs.					
18. Consumers are not generally happy with the unified medical prescription form.					
19. Consumers have been actively requesting generic substitutions for brand name drugs.					
20. Consumers have accepted most of the substitution suggestions offered by the pharmacy.					
21. Some consumers/patients still show up with the old prescription form.					
22. The number of generic dispensing at my pharmacy has increased considerably since the implementation of the policy.					
23. There was an initial peak in generic dispensing following the implementation of the policy that was subsequently attenuated.					
24. The overall patient expenditure on medicine has decreased since the implementation of the policy.					
25. There has been a noticeable shift in pharmaceutical companies’ promotional strategies targeting pharmacists.					
26. The policy is creating conflicts between the physician, the pharmacist and the patient.					

<u>Section IV: Perceived barriers and facilitators to the implementation of the generic drug substitution policy</u>

1. From your experience, what are the top three barriers to the correct (i.e., as intended by the ministry) implementation of the generic drug substitution policy

____
____
____
____
____
____
____

2. From your experience, what strategies should be implemented to ensure the generic drug substitution policy achieves its intended purposes?

____
____
____
____
____
____
____

Thank you for completing the survey

Please insert the completed survey in the envelope before returning it to the research team member

الرقم التسلسلي: __________

دراسة إحصائية

شكراً على تخصيص وقتكم وجهدكم للمشاركة في هذه الدراسة الإحصائية.
تنقسم هذا الاستمارة إلى أربعة فقرات قصيرة.
يقدّر الوقت المطلوب لاستكمال هذه الاستمارة بحوالي 20 دقيقة (أو أقل).
لا يطلب منكم في أي قسم أو خانة من هذه الاستمارة كتابة الاسم أو التوقيع.

أولاً: الخصائص الديموغرافية

يرجى استكمال البيانات المطلوبة التالية من خلال وضع علامة "x" في الخانة التي تعبر عن الاختيار الأكثر دقة (اختيار خانة واحدة فقط لكل بند).

المتغيرات	
**القضاء**	☐ بيروت<br>☐ جبل لبنان<br>☐ شمال لبنان<br>☐ البقاع<br>☐ جنوب لبنان<br>☐ النبطية
**الجنس**	☐ ذكر<br>☐ أنثى
**الفئة العمرية**	☐ 20-30<br>☐ 31-40<br>☐ 41-50<br>☐ 51-60<br>☐ 61 وما فوق
**الصفة المهنية**	☐ صيدلي<br>☐ فني صيدلة<br>☐ فئة أخرى (يرجى تحديد ذلك):<br>__________
**ملكية الصيدلية تعود لك:**	☐ نعم<br>☐ كلا
**الدولة التي حصلت فيها على الشهادة**	☐ لبنان<br>☐ أمريكا الشمالية<br>☐ أوروبا الغربية<br>☐ أوروبا الشرقية (بما في ذلك روسيا)<br>☐ غيرها (يرجى تحديد الدولة):<br>__________
**هل تحمل (تحملين) شهادة أو مؤهلات عليا في الصيدلة (دكتوراة صيدلة PharmD)؟**	☐ نعم<br>☐ كلا

**عدد سنوات الخبرة في العمل بصفة صيدلي أو فني صيدلة**	يرجى تحديد عدد السنوات: ________________
**هل تلقيت أي تدريب خلال دراستك عن الأدوية ذات العلامة التجارية المسجلة مقارنة بأدوية الجينيريك (غير محدودة الملكية)؟**	☐ نعم<br>☐ كلا

ثانياً: السلوكيات تجاه استبدال الأدوية بالجينيريك والوصفة الطبية الموحدة

في هذه الدراسة، نعني بكلمة "علامة تجارية مسجلة" أي علامة تجارية مسجلة لدواء، في حين نعني بالصفة "الجينيريك " أي أدوية ذات صفة علاجية متماثلة لها غير محدودة الملكية.

يرجى تحديد مستوى موافقتكم أو رفضكم لكل من العبارات التالية من خلال وضع علامة "**x**" في الخانة التي تعبر بالشكل الأكثر دقة عن موقفكم.

العبارة	أعترض بشدة	أعترض	حيادي	أوافق	أوافق بشدة
**1. أؤيد استبدال كافة الأدوية ذات العلامة التجارية المسجلة بأدوية الجينيريك (غير محدودة الملكية) متى ما توفر ذلك.**					
**2. يجوز للصيدلي أن يستبدل الأدوية ذات العلامة التجارية المسجلة بأدوية الجينيريك من دون الحاجة إلى استحصال إذن من الطبيب الذي وصف الدواء.**					
**3. يجوز للصيدلي أن يعتمد على مندوبي شركات الأدوية للاطلاع على الوصفات البديلة للأدوية.**					
**4. أؤيد تطبيق الوصفة الطبية الموحدة.**					
**5. إن الوصفة الطبية الموحدة تساعد على الترويج لاستخدام أدوية الجينيريك في لبنان.**					
**6. إن الوصفة الطبية الموحدة تساعد على تحديد الأطباء الأكثر تأثراً بمندوبي شركات الأدوية.**					
**7. إن الوصفة الطبية الموحدة تساعد على تنظيم وضبط شركات صناعة الأدوية .**					
**8. يجب الإبقاء على خيار "غير قابل للاستبدال" في النموذج، وهو خيار يسمح للطبيب بأن يفرض على الصيدلي عدم استبدال الدواء كما يرد في الوصفة الطبية، في حال وضع علامة عليه.**					
**9. أشعر بالرضا تجاه التصميم العام لنموذج الوصفة الطبية الموحدة.**					
**10. إن نموذج الوصفة الطبية الموحدة سهل الاستخدام.**					

ثالثاً: الممارسات والتجارب فيما يتعلق بتطبيق سياسة استبدال الأدوية بالجينيريك

يرجى تحديد مستوى موافقتكم أو رفضكم لكل من العبارات التالية من خلال وضع علامة "**x**" في الخانة التي تعبر بالشكل الأكثر دقة عن موقفكم.

العبارة	أعترض بشدة	أعترض	حيادي	أوافق	أوافق بشدة
**1. هناك التزام من طرف الصيادلة تجاه القوانين التي تحكم الوصفة الطبية الموحدة.**					
**2. قمت باستبدال الأدوية ذات العلامة التجارية المسجلة بأدوية الجينيريك في معظم الوصفات التي قمت بتسلمها وتسليمها في الصيدلية (باستثناء الوصفات التي تنص على خيار غير قابل للاستبدال).**					
**3. إن نظام التسعير القائم حالياً لا يشجعني على استبدال الأدوية ذات العلامة التجارية المسجلة بأدوية الجينيريك.**					
**4. يتأثر قراري بخصوص استبدال أي دواء على تقديري للحالة الاقتصادية والاجتماعية للمريض/ الزبون.**					
**5. يتأثر قراري بخصوص خيار الاستبدال على المعلومات التي أحصل عليها من مندوبي شركات الأدوية.**					
**6. منذ تطبيق قانون الوصفة الطبية الموحدة، أصبحت أشعر بالقدرة على الحديث مع الزبائن والمرضى حول استبدال الأدوية بالجينيريك.**					
**7. لا أصف استبدال الأدوية بالجينيريك إلا في حال بادر المريض أو الزبون بطلب ذلك.**					
**8. أحياناً أقوم باستشارة الطبيب، وتحديداً عندما أشعر بأن خيار "غير قابل للاستخدام" الذي وصفه غير مبرر.**					
**9. ألتزم بقائمة وزارة الصحة العامة التي تم إقرارها فيما يتعلق بأدوية الجينيريك التي يمكن استبدالها.**					
**10.هناك التزام من طرف الأطباء تجاه القوانين التي تحكم الوصفة الطبية الموحدة.**					
**11. هناك بعض الإشكاليات الفنية/ التقتية في تطبيق الوصفة الطبية الموحدة.**					
**12. لا توجد توجيهات موحّدة وواضحة فيما يتعلق بكيفية استخدام النماذج المختلفة للوصفة الطبية.**					
**13. يلجأ الأطباء بشكل عام إلى إساءة استخدام خيار "غير قابل للاستبدال".**					

العبارة	أعترض بشدة	أعترض	حيادي	أوافق	أوافق بشدة
14. تجري وزارة الصحة العامة كشوفات دورية للتحقق من نماذج الوصفات التي تتسلمها الصيدلية.					
15. يتوافرمعظم الأحيان لدى الصيدلية مخزون من أدوية الجينيريك، التي تقرها وزارة الصحة العامة.					
16. إن القائمة الوطنية الصادرة عن وزارة الصحة العامة لأدوية الجينيريك القابلة للاستبدال محدثة، ويسهل الحصول عليها واستخدامها.					
17. يعبر المرضى والزبائن بشكل عام عن نظرة سلبية تجاه أدوية الجينيريك.					
18. لا يبدو أن المرضى راضون و/أو سعيدون بشكل عام بتطبيق الوصفة الطبية الموحدة.					
19. يبادر المرضى والزبائن بشكل ملحوظ بطلب استبدال الأدوية ذات العلامات التجارية المسجلة بأدوية الجينيريك.					
20. يتقبل المرضى والزبائن بشكل عام معظم الاستبدالات التي نقترحها عليهم في الصيدلية.					
21. لا يزال بعض المرضى والزبائن يعتمدون على النموذج القديم للوصفة الطبية.					
22. لقد ازداد عدد تسليم أدوية الجينيريك (غير محدودة الملكية) في الصيدلية عندي بشكل ملحوظ منذ البدء بتطبيق سياسة استبدال الأدوية بالجينيريك.					
23. عند بدء تطبيق سياسة استبدال الأدوية بالجينيريك ، حصل ارتفاع حاد في تسليمات أدوية الجينيريك في الصيدلية، لكن سرعان ما اعتدل هذا العدد تدريجيا.					
24. تراجع إجمالي إنفاق المرضى على الأدوية منذ تطبيق سياسة استبدال الأدوية بالجينيريك.					
25. حدث تغير ملحوظ في استراتيجيات الترويج التي تعتمدها شركات الأدوية تجاه الصيادلة.					
26. إن سياسة استبدال الأدوية بالجينيريك تؤدي إلى بعض الإشكالات ما بين الطبيب والصيدلي والمريض.					

رابعاً: تقديركم للعوامل والأمور التي تعيق أو تسهل عملية تطبيق سياسة استبدال الأدوية ذات العلامة التجارية المسجلة بأدوية الجينيريك

1. من خلال تجربتكم، ما هي العوامل الثلاث الأهم التي تعيق تطبيق سياسة استبدال الأدوية ذات العلامة التجارية المسجلة بأدوية الجينيريك(أي كما ترغب الوزارة)؟

____
____
____
____
____
____
____
____
____

2. من خلال تجربتكم، ما هي الاستراتيجيات التي يجب اعتمادها لضمان تطبيق سياسة استبدال الأدوية ذات العلامة التجارية المسجلة بأدوية الجينيريك بشكل سليم يحقق الأهداف التي تم وضعها لأجلها؟

____
____
____
____
____
____
____
____
____

شكراً على مشاركتكم القيمة

يرجى وضع الاستمارة المكتملة في الظرف المرفق قبل إعادتها إلى الفرد المعني من فريق البحث

Interview Guide

Introduction

- Interviewer to introduce himself/herself to the respondent
- Remind respondent of the purpose of the interview and the projected length
- Remind respondent of the voluntary nature of the study and their right to withdraw at any time without any consequences
- Confirm participant's verbal consent to participate in the interview
- Ask respondent for his/her verbal consent with respect to audio recording the interview and remind him/her that his/her statements will be kept confidential at all times.

Purpose of the interview

The Ministry of Public Health, along with other associated ministries, has introduced the unified medical prescription form starting August 10th, 2015. During this interview, I would like to elicit your input on the unified medical prescription and reflect on your experience with implementing it so far.

Examples of interview questions

<table>
<tr><th>Main Question</th><th>Additional Question</th><th>Clarifying questions</th></tr>
<tr><td>Can you tell me more about the generic drug substitution policy and its intended purposes?</td><td>- Do you support its implementation?</td><td rowspan="3">- Can you expand a little on this?

- Can you tell me anything else?

- Can you give me some examples?</td></tr>
<tr><td>Can you tell me about the challenging problems you've encountered since implementing the unified medical prescription?</td><td>- What gave rise to them?
- How can they be overcome?</td></tr>
<tr><td>Generally speaking, are users satisfied with the generic drug substitution policy? (Patients? Physicians? Pharmacists?)</td><td>- Do you know of any groups who are dissatisfied with its implementation? Why?</td></tr>
</table>

Conclude the interview

- When interviewers feel that all topics have been discussed, ask the respondent if he/she has anything to add.
- Thank respondent for participating and explain how the rest of the project will proceed

دليل إجراء المقابلات

التقديم

- يبدأ الشخص الذي يجري المقابلة بالتعريف عن نفسه للشخص موضوع المقابلة (المشارك)
- تذكير المشارك بهدف المقابلة والمدة الزمنية التي يتوقع أن تستغرقها المقابلة
- تذكير المشارك بأن المشاركة في هذه الدراسة اختيارية طوعية بالكامل، وأن له(ا) كامل الحق بالانسحاب من المشاركة في أي وقت كان، من دون أن يكون لهذا الانسحاب أي عواقب من أي نوع
- تأكيد الموافقة الطوعية للمشارك شفاهياً قبل البدء بالمقابل
- الاستفسار من المشارك عن موافقته(ا) على التسجيل الصوتي للمقابلة، مع التذكير والتأكيد بأن جميع إجاباته وتصريحاته ستخضع لأعلى معايير السرية والخصوصية بجميع الأوقات

الهدف من المقابلة

استحدثت وزارة الصحة العامة بالتنسيق مع الوزارات الأخرى المعنية نموذج الوصفة الطبية الموحدة وبدأ العمل بها من 10 آب/أغسطس 2015. نرغب من خلال هذه المقابلة باستطلاع آرائكم حول هذا النموذج (الوصفة) وتقييمكم لتجربتكم في استخدامها منذ ذلك التاريخ وحتى اليوم.

أمثلة عن أسئلة المقابلة

الأسئلة الرئيسية	أسئلة إضافية	أسئلة بهدف الاستيضاح مزيداً
هل يمكن أن تخبرنا المزيد عن سياسة استبدال الأدوية بالجينيريك (الأدوية المكافئة نوعياً بعلامة تجارية أخرى) والهدف منها؟	- هل تدعمون تنفيذ هذه السياسة؟	- هل يمكن التوسع قليلاً وإعطاءنا مزيداً من التفاصيل بهذا الخصوص؟<br>- هل هناك أي شيء ترغب بقوله أيضاً / إضافته؟<br>- هل من الممكن إعطاءنا بعض الأمثلة؟
هل يمكن أن تخبرنا عن أبرز المشاكل التي تواجهونها منذ بداية العمل باستخدام نموذج الوصفة الطبية الموحدة؟	- برأيكم، ما الذي أدى إلى حصول هذه المشاكل؟<br>- كيف يمكن حل هذه المشاكل أو مواجهتها والحدّ منها؟	
بشكل عام، هل يبدي المستخدمون رضاهم عن سياسة استبدال الأدوية بالجينيريك ؟ (المرضى؟ الأطباء؟ الصيادلة؟)	- هل لديكم علم بوجود أي فئة أو مجموعة ممن هم غير راضين عن تنفيذ هذه السياسة؟ لماذا برأيكم؟	

إنهاء المقابلة

- عندما يشعر من يجري المقابلة أنه تمت مناقشة جميع المواضيع والقضايا المطلوبة، يتم سؤال المشارك عمّا إذا كان لديه أي إضافات أو ملاحظات أخرى يودّ قولها
- توجيه الشكر للمشارك وتقديم صورة عامة عن الكيفية التي سيسير عليها المشروع
