# Supplementary material for: Pharmacists’ views and reported practices in relation to a new generic drug substitution policy in Lebanon: a mixed methods study
Source: Implement Sci. 2017 Feb 17;12:23. doi: 10.1186/s13012-017-0556-1 (PMC5316154; doi:10.1186/s13012-017-0556-1)
Supplement: Additional file 2: — Results of the full Likert scale. (PDF 275 kb) [file 13012_2017_556_MOESM2_ESM.pdf]

Additional file 2: Results of the Full Likert Scale

Scales and corresponding items	Mean	Std. Deviation	Strongly Disagree N (%)*	Disagree N (%)*	Neutral N (%)*	Agree N (%)*	Strongly agree N (%)*
**Attitude towards Generic Drug Substitution (Cronbach's alpha= 0.553)**	**3.78**	**0.85**	**8 (2.6)**	**41 (13.5)**	**35 (11.6)**	**145 (47.8)**	**74 (24.4)**
1. I support generic substitution for all brand name drugs for which generics are available	3.55	1.01	4 (2.6)	26 (17.1)	25 (16.4)	77 (50.7)	20 (13.2)
2. It is acceptable that pharmacists perform generic substitution without obtaining permission from the prescribing physician	4.01	1.03	4 (2.6)	15 (9.9)	10 (6.6)	68 (45)	54 (35.8)
**Attitude towards Unified Medical Prescription (Cronbach's alpha= 0.529)**	**3.16**	**0.77**	**65 (10.7)**	**145 (23.8)**	**122 (20.0)**	**178 (29.2)**	**99 (16.3)**
1. I support the implementation of the unified medical prescription	2.94	1.22	21 (14.1)	37 (24.8)	36 (24.2)	40 (26.8)	15 (10.1)
2. The unified medical prescription helps promote the use of generic drugs in Lebanon	3.09	1.25	19 (12.7)	34 (22.7)	31 (20.7)	47 (31.3)	19 (12.7)
3. The unified medical prescription helps identify physicians who are influenced by medical representatives	3.89	1.09	5 (3.4)	15 (10.1)	22 (14.8)	56 (37.6)	51 (34.2)
4. The unified medical prescription helps regulate the pharmaceutical industry	2.83	1.2	20 (13.2)	59 (32.5)	33 (21.9)	35 (23.2)	14 (9.3)
**Layout/structure of Form (Cronbach's alpha= 0.705)**	**3.16**	**0.77**	**30 (9.9)**	**58 (19.1)**	**78 (25.7)**	**127 (41.8)**	**11 (3.6)**
1. I am satisfied with the overall layout of the form	2.89	1.09	19 (12.5)	38 (25)	40 (26.3)	51 (33.6)	4 (2.6)
2. The form is user-friendly	3.32	1.01	11 (7.2)	20 (13.2)	38 (25)	76 (50)	7 (4.6)
**Pharmacist Practices (Cronbach's alpha= 0.566)**	**3.30**	**0.64**	**46 (6.1)**	**140 (18.5)**	**193 (25.6)**	**293 (38.8)**	**83 (11.0)**
1. Pharmacists are adhering to the laws that govern the unified medical prescription.	2.11	1.18	7 (4.6)	22 (14.5)	33 (21.7)	68 (44.7)	22 (14.5)
2. I have substituted brand drugs for generic equivalents in most of the prescriptions I have dispensed (excluding	3.04	1.14	14 (9.3)	39 (25.8)	38 (25.2)	47 (31.1)	13 (8.6)

Scales and corresponding items	Mean	Std. Deviation	Strongly Disagree N (%)*	Disagree N (%)*	Neutral N (%)*	Agree N (%)*	Strongly agree N (%)*
those prescriptions where the "NS" option was ticked)							
3. I feel empowered to speak to patients about generic drug substitution since the implementation of the policy	2.99	1.01	10 (6.6)	40 (26.5)	51 (33.8)	42 (27.8)	8 (5.3)
4. I sometimes consult with the physician when I feel the "NS" option is used unjustifiably	3.32	1.18	12 (7.9)	30 (19.9)	27 (17.9)	61 (40.4)	21 (13.9)
5. I have been adhering to the Ministry of Public Health's (MOPH) agreed list of substitutable generic drugs	3.65	0.85	3 (2.0)	9 (6.0)	44 (29.3)	75 (50)	19 (12.7)
**MOPH Responsiveness (Cronbach's alpha= 0.324)**			**47(7.8)**	**128 (21.3)**	**173 (28.8)**	**215 (35.8)**	**38 (6.3)**
1. The MOPH performs regular audits on the prescription forms collected by the pharmacy	3.21	1.13	17 (11.3)	19 (12.7)	43 (28.7)	58 (38.7)	13 (8.7)
2. Generic drug equivalents approved by the MOPH are almost always in stock	3.56	0.81	2 (1.3)	14 (9.3)	42 (28.0)	82 (54.7)	10 (6.7)
3. The MOPH's national list of substitutable generic drugs is updated, accessible and easy to use	3.18	1	8 (5.3)	30 (19.7)	51 (33.6)	53 (34.9)	10 (6.6)
4. The existing price structure discourages me from performing generic drug substitution [r]	2.51	1.01	5 (3.4)	22 (14.8)	37 (24.8)	65 (43.6)	20 (13.4)
**Functionality of Form (Cronbach's alpha= 0.452)**			**237 (31.4)**	**334 (44.3)**	**88 (11.6)**	**66 (8.8)**	**29 (3.8)**
1. Physicians are adhering to the laws that govern the unified medical prescription form	2.11	1.18	57 (38)	51 (34)	19 (12.7)	15 (10)	8 (5.3)
2. There are technical problems with the implementation of the unified medical prescription form [r]	2.14	0.9	2 (1.3)	15 (9.9)	16 (10.5)	88 (57.9)	31 (20.4)
3. There are no clear guidelines on how to use the forms [r]	2.36	1.06	7 (4.6)	20 (13.2)	21 (13.8)	77 (50.7)	27 (17.8)
4. Physicians in general are abusing the "NS" option [r]	1.83	0.99	5 (3.3)	7 (4.6)	12 (7.9)	60 (39.7)	67 (44.4)

Scales and corresponding items	Mean	Std. Deviation	Strongly Disagree N (%)*	Disagree N (%)*	Neutral N (%)*	Agree N (%)*	Strongly agree N (%)*
5. Some patients still show up with the old prescription form [r]	2.03	1.08	7 (4.7)	9 (6)	20 (13.4)	58 (38.9)	55 (36.9)
**Consumer Acceptance (Cronbach's alpha= 0.472)**			**73 (12.1)**	**231 (38.3)**	**130 (21.6)**	**143 (23.7)**	**26 (4.3)**
1. Consumers generally express negative attitudes towards generic drugs [r]	2.61	1.13	8 (5.3)	31 (20.7)	28 (18.7)	60 (40.0)	23 (15.3)
2. Consumers are not generally happy with the unified medical prescription [r]	2.2	0.89	2 (1.3)	11 (7.2)	34 (22.4)	74 (48.7)	31 (20.4)
3. Consumers have been actively requesting generic substitutions for brand name drugs	2.89	1.06	12 (7.9)	50 (33.1)	39 (25.8)	43 (28.5)	7 (4.6)
4. Consumers have accepted most of the substitution suggestions offered by the pharmacy	3.1	1.06	7 (4.7)	47 (31.3)	29 (19.3)	58 (38.7)	9 (6.0)
**Relation with Drug Companies (Cronbach's alpha= 0.629)**	**2.76**	**0.92**	**36 (12.0)**	**104 (34.7)**	**68 (22.7)**	**81 (27.0)**	**11(3.7)**
1. My drug substitution choice is influenced by information from medical drug representatives	2.6	1.01	19 (12.6)	60 (39.7)	36 (23.8)	34 (22.5)	2 (1.3)
2. It is acceptable that pharmacists rely on medical drug representatives to learn about alternative drug substitutions	2.91	1.15	17 (11.4)	44 (29.5)	32 (21.5)	47 (31.5)	9 (6.0)
**Outcome of the Policy (Cronbach's alpha= 0.511)**	**2.67**	**0.61**	**95 (12.5)**	**282 (37.2)**	**188 (24.8)**	**161 (21.2)**	**32 (4.2)**
1. The number of generic dispensing at my pharmacy has increased considerably since the implementation of the policy	2.59	0.97	16 (10.5)	65 (42.8)	39 (25.7)	30 (19.7)	2 (1.3)
2. There was an initial peak in generic dispensing following the implementation of the policy that was subsequently attenuated	2.72	1.02	14 (9.2)	59 (38.8)	39 (25.7)	35 (23)	5 (3.3)
3. The overall patient expenditure on medicine has decreased since the implementation of the policy	2.86	1.09	12 (7.9)	57 (37.5)	31 (20.4)	44(28.9)	8 (5.3)
4. There has been a noticeable shift in pharmaceutical companies' promotional	2.97	1.05	10 (6.6)	43 (28.3)	53 (34.9)	34 (22.4)	12 (7.9)

Scales and corresponding items	Mean	Std. Deviation	Strongly Disagree N (%)*	Disagree N (%)*	Neutral N (%)*	Agree N (%)*	Strongly agree N (%)*
strategies targeting pharmacists							
5. The policy is creating conflicts between the physician, the pharmacist and the patient [r]	3.77	1.09	5 (3.3)	18 (12.0)	26 (17.3)	58 (38.7)	43 (28.7)

*All percentages have been rounded to one decimal point
[r] reverse scores
