# Supplementary material for: Pharmacists’ views and reported practices in relation to a new generic drug substitution policy in Lebanon: a mixed methods study
Source: Implement Sci. 2017 Feb 17;12:23. doi: 10.1186/s13012-017-0556-1 (PMC5316154; doi:10.1186/s13012-017-0556-1)
Supplement: Additional file 3: — Questionnaire distribution by governorate and response rates. (PDF 84 kb) [file 13012_2017_556_MOESM3_ESM.pdf]

Additional file 3: Questionnaire distribution by governorate and response rates

**Governorate**	**Number of pharmacies approached**	**Number of respondents**	**Percent response rate***
Beirut	40	31	78%
Bekaa	34	22	65%
Mount Lebanon	32	27	84%
North	37	29	78%
Nabatiye	31	21	68%
South Lebanon	30	23	77%
**Total**	**204**	**153**	**75%**
*All percentages have been rounded to the nearest one			
