# Supplementary material for: Pharmacists’ views and reported practices in relation to a new generic drug substitution policy in Lebanon: a mixed methods study
Source: Implement Sci. 2017 Feb 17;12:23. doi: 10.1186/s13012-017-0556-1 (PMC5316154; doi:10.1186/s13012-017-0556-1)
Supplement: Additional file 4: — ANOVA and T tests results. (PDF 314 kb) [file 13012_2017_556_MOESM4_ESM.pdf]

Additional file 4: ANOVA and T-tests results *

	**Pharmacist attitude towards generic drug substitution**	**Pharmacist attitude towards unified medical prescription**	**Layout/ structure of form**	**Outcome of policy**	**Relation with drug company**	**Functionality of form**	**Consumer acceptance**	**Pharmacist practices**	**MOPH Responsiveness**
	Mean** (SD)	Mean (SD)	Mean (SD)	Mean (SD)	Mean (SD)	Mean (SD)	Mean (SD)	Mean (SD)	Mean (SD)
**Gender**									
Male	**3.96 (0.85)**	3.27 (0.81)	3.13 (0.83)	2.91 (0.65)	2.70 (0.94)	2.53 (0.56)	2.76 (0.64)	3.28 (0.65)	3.13 (0.54)
N***	74	72	75	74	73	75	74	74	71
Female	3.57 (0.81)	3.03 (0.69)	3.06 (1.02)	3.02 (0.62)	2.8 (0.93)	2.41 (0.41)	2.63 (0.65)	3.29 (0.63)	3.08 (0.60)
N	72	67	72	71	70	67	68	71	69
P-value	***0.005***	0.059	0.614	0.291	0.488	0.155	0.229	0.912	0.603
**Postgraduate Qualification (PharmD)**									
**Yes**	3.84 (0.85)	3.05 (0.82)	2.74 (1.05)	3.08 (0.65)	2.78 (0.98)	2.39 (0.50)	2.65 (0.65)	3.37 (0.64)	3.02 (0.64)
N	56	55	57	56	56	56	56	57	56
**No**	3.74 (0.86)	3.22 (0.73)	**3.30 (0.76)**	2.90 (0.63)	2.73 (0.90)	2.51 (0.48)	2.74 (0.66)	3.25 (0.64)	3.16 (0.53)
N	92	86	92	91	90	87	88	90	87
P-value	0.491	0.192	**0.*001***	0.105	0.784	0.161	0.438	0.272	0.146
**Received teaching about generic versus brand name drugs**									
Yes	3.74 (0.88)	**3.25 (0.78)**	2.98 (0.95)	3.00 (0.6)	2.78 (0.95)	2.47 (0.53)	2.69 (0.67)	**3.41 (0.63)**	3.08 (0.61)
N	84	81	84	83	83	80	81	83	79

	**Pharmacist attitude towards generic drug substitution**	**Pharmacist attitude towards unified medical prescription**	**Layout/ structure of form**	**Outcome of policy**	**Relation with drug company**	**Functionality of form**	**Consumer acceptance**	**Pharmacist practices**	**MOPH Responsiveness**
	Mean** (SD)	Mean (SD)	Mean (SD)	Mean (SD)	Mean (SD)	Mean (SD)	Mean (SD)	Mean (SD)	Mean (SD)
No	3.88 (0.77)	2.93 (0.70)	**3.41 (0.81)**	2.87 (0.48)	2.70 (0.85)	2.43 (0.36)	2.7 (0.60)	2.96 (0.50)	3.16 (0.45)
N	67	62	68	67	65	66	66	67	66
P-value	0.17	***0.035***	***0.013***	0.232	0.681	0.576	0.892	***<0.001***	0.441
**Governorate******									
Beirut	3.66 (0.72)	3.06 (0.75)	2.97 (0.88)	2.92 (0.60)	2.83 (0.88)	2.49 (0.55)	2.44 (0.53)a	3.37 (0.60)	3.01 (0.55)a
N	31	28	31	31	30	30	29	30	31
Mount Lebanon	3.89 (0.82)	2.94 (0.53)	3.24 (0.93)	2.98 (0.59)	2.37 (0.79)	2.42 (0.66)	2.86 (0.54)	3.04 (0.53)	3.27 (0.48)
N	22	22	23	23	23	22	22	23	23
North Lebanon	3.73 (1.00)	3.48 (0.92)	3.13 (0.80)	2.93 (0.62)	2.90 (0.88)	2.3852 (0.44002)	2.51 (0.59)b	3.26 (0.70)	3.04 (0.58)b
N	28	24	28	27	26	27	27	28	27
Beqaa	3.96 (1.01)	3.18 (0.84)	3.21 (0.95)	2.88 (0.60)	2.58 (1.01)	2.45 (0.35)	2.82 (0.67)	3.17 (0.57)	3.03 (0.46)c
N	26	26	26	26	26	26	26	26	24
South Lebanon	3.67 (0.68)	3.26 (0.68)	3.39 (0.80)	3.14 (0.70)	3.11(0.89)	2.56 (0.29)	**3.09 (0.69) a, b**	3.6 (0.64734)	**3.55 (0.58)a, b, c, d**
N	23	22	23	22	22	21	22	22	21
Nabatieh	3.79 (0.85)	3.00 (0.72)	2.67 (1.11)	3.07 (0.79)	2.74 (1.01)	2.51 (0.62)	2.61 (0.71)	3.38 (0.69)	2.80 (0.56)d
N	21	21	21	21	21	20	21	21	19
P-value	0.770	0.171	0.128	0.739	0.100	0.856	***0.003***	0.059	***<0.001***

	Pharmacist attitude towards generic drug substitution	Pharmacist attitude towards unified medical prescription	Layout/ structure of form	Outcome of policy	Relation with drug company	Functionality of form	Consumer acceptance	Pharmacist practices	MOPH Responsiveness
	Mean** (SD)	Mean (SD)	Mean (SD)	Mean (SD)	Mean (SD)	Mean (SD)	Mean (SD)	Mean (SD)	Mean (SD)
**Years of experience**									
Less than 10 years	3.76 (0.83)	3.16 (0.68)	2.98 (0.93)	3.02 (0.60)	2.75 (0.93)	2.42 (0.48)	2.65 (0.61)	3.21 (0.62)	3.00 (0.58)
N	84	81	84	83	83	80	81	83	79
More than 10 years	3.80 (0.87)	3.15 (0.87)	3.26 (0.89)	2.93 (0.69)	2.77 (0.93)	2.53 (0.50)	2.78 (0.69)	**3.41 (0.64)**	**3.25 (0.54)**
N	67	62	68	67	65	66	66	67	66
P-value	0.794	0.915	0.061	0.388	0.885	0.182	0.223	***0.049***	***0.009***

* Results are shown for only the variables with significant association to any of the scales

** Scores range from 1 to 5 for each scale, with higher scores signifying higher agreement to the item being assessed.

*** N= sample size

****ANOVA test was conducted, values that have the same letter denote significant differences
