# Supplementary material for: Pharmacists’ views and reported practices in relation to a new generic drug substitution policy in Lebanon: a mixed methods study
Source: Implement Sci. 2017 Feb 17;12:23. doi: 10.1186/s13012-017-0556-1 (PMC5316154; doi:10.1186/s13012-017-0556-1)
Supplement: Additional file 5: — Barriers and strategies to facilitate implementation of the generic drug substitution policy, as perceived by respondents. (PDF 193 kb) [file 13012_2017_556_MOESM5_ESM.pdf]

Additional file 5: Barriers and strategies to facilitate implementation of the generic drug substitution policy, as perceived by respondents

| Level | Perceived Barriers | Suggested strategies to overcome barriers |
|---|---|---|
| **Physicians** | Overuse by physicians of the 'non-substitutable' option for "unjustified" reasons including for drugs that are not available in the market | - Cancel the NS option<br>- Educate physicians on the purpose of the NS option and the need to respect its use<br>- Encourage physicians to take into account the economic condition of patient when prescribing the medicine<br>- Establish a limit on the minimum permissible number of NS that can be written by a physician per month. |
| | Continued use of the old form by physicians, especially for patients who are uninsured or who have private insurance<br><br>Physicians charging patients for the new form | - Persuade physicians to use the new form and circulate reminders<br>- Prosecute physicians who refuse to use the new form<br>- Facilitate availability and access to form<br>- Distribute form free of charge<br>- Prosecute physicians who charge patients for the form. |
| | Physicians' interactions with the pharmaceutical industry | - Restrict interactions between physicians and pharmaceutical companies<br>- Impose fines on doctors who accept bribery to prescribe certain drugs<br>- Raise awareness among physicians on the effects of physician-industry interactions on their behaviors |
| | Insufficient awareness of physicians of the importance of applying the policy as well as the rules that govern it | - Organize seminars and conferences to all concerned parties including physicians and hospital directors, to explain the goals of the policy and clarify the duties, rights and expectations from each party. |
| | Errors and omissions when filling prescription form as well as incorrect stamping of form | - Simplify the form layout and requirements |
| **Pharmacists** | Lack of support by pharmacists for the new policy<br><br>Poor commitment of pharmacists to the new prescription form with continued acceptance of old form | - Organize seminars and conferences to all concerned parties including pharmacists to explain the goals of the policy and clarify the duties, rights and expectations from each party<br>- Mainstream role of pharmacist to be recognized by all physician<br>- Empower pharmacists and grant them the right to perform drug substitution if deemed necessary without referring to physician<br>- Issue a full list of over-the-counter drugs by the MOPH that can be disbursed by |

| Level | Perceived Barriers | Suggested strategies to overcome barriers |
|---|---|---|
| | | pharmacists to the patient without a prescription form.<br>- Clarify the law to regulate the operations of replacement medication. |
| | Lack of incentives to perform generic substitution | - Provide compensation for pharmacists for their losses because of the reduction in the prices of medicines.<br>- Establish incentive systems to promote generic substitution |
| | Administrative burden with processing the form with subsequent rejections | - Reduce administrative burden and provide some flexibility when processing the form |
| | Pharmacists' lack of awareness of the existence of a national list of approved substitutable drugs on the MOPH website | - Raise awareness of pharmacists and involve them in the decision-making process |
| **Consumers** | Poor awareness and education of consumer about generic drugs and the unified medical prescription | - Leverage on different media channels to promote the new policy |
| | Resistance of consumers to generic drugs and generic substitution | - Conduct awareness campaigns to raise the awareness of consumer about generic drugs and the unified medical prescription.<br>- Third party payers to promote generic substitution by disbursing only generic equivalents of brand name drugs |
| | Lack of trust in pharmacists and preference to purchase the exact medication prescribed by physicians | - Through emphasizing the important role of pharmacist in ensuring the quality and safety of medications |
| **Organizational** | Poor adherence of hospitals and dispensaries to the new policy | - Ensure the full commitment of hospitals and dispensaries to the new policy |
| | Some hospitals charge patients for the new form | - Prosecute entities who refrain from its proper use including those that charge patients for the form. |
| **System** | Absence of mechanisms to monitor prescribing patterns | - Computerize prescription forms to allow ongoing and timely surveillance<br>- Adopt magnetic cards to facilitate monitoring of drug prescription patterns |
| | Poor enforcement systems in place | - Periodic inspections of physicians and pharmacists and dispensaries by the ministry of public health to ensure they are abiding by the policy<br>- Prosecution of, and imposition of fines on entities that violate the rules |
| | Challenging financial and economic situations that affect some patients' ability to afford consultation fees whenever they want a medication | - Restructure the health care system to ensure free medical services to all citizens<br>- Subsidize consultation fees for poor<br>- Ensure funds to aid the sick |
| | Lack of universal health coverage which may hinder consumer access to medication | - Accelerate progress to universal health coverage |

| Level | Perceived Barriers | Suggested strategies to overcome barriers |
|---|---|---|
| | | - Expand National Social Security Funds (NSSF) beneficiaries (respondents claimed that doctors/organizations tend to use new form with NSSF patients) |
| | Delays from insurance companies in processing payment of bills related to medicines disbursed according to new prescription form | - NSSF to accelerate processing payment of bills related to drugs disbursed according to prescription form. |
| | Lack of collection of forms by the MOPH resulting in piling up of paperwork at pharmacies | - Switch to electronic version that is simple, less time consuming, printable and for free |
| | Price of several brand name drugs is similar to their generic alternatives, thus discouraging generic substitution | - Impose appropriate pricing policies that create a competitive market for generic drugs |
| | Poor trust of pharmacists, physicians and patients in quality and effectiveness of all generic drugs available in market | - MOPH to ensure quality of marketed generic drugs through interventions such as: (1) mandatory quality and bioequivalence testing of generic drugs; (2) requirement for approval of generic drugs by international organizations such as FDA, EMA, and Canadian ones |
| | Insufficient quantities of alternative medicines which hinder their ability to perform substitutions | - Expand the list of alternative generic medicines<br>- Do not register new brands without specifying number of equivalent generics for that brand |
| | Prescription form does not cover army and dental services | - Expand the scope of the policy |
| | Absence of regulations on pharmaceutical industry's interactions with health care professionals | - Restrictive policies preventing certain types of interactions and limiting others<br>- Disclosure of interactions to the public |
